# Effect of dose reduction on image quality and diagnostic performance in coronary computed tomography angiography

**DOI:** 10.1007/s10554-012-0096-3

**Published:** 2012-09-22

**Authors:** Noortje van der Bijl, Raoul M. S. Joemai, Bart J. A. Mertens, Albert de Roos, Wouter J. H. Veldkamp, Jeroen J. Bax, Joanne D. Schuijf, Jacob Geleijns, Lucia J. M. Kroft

**Affiliations:** 1 Department of Radiology, C2-S, Leiden University Medical Center, Albinusdreef 2, 2333 ZA Leiden, The Netherlands; 2 Department of Medical Statistics and Bioinformatics, Leiden University Medical Center, Einthovenweg 20, 2333 ZC Leiden, The Netherlands; 3 Department of Cardiology, Leiden University Medical Center, Albinusdreef 2, 2333 ZA Leiden, The Netherlands

**Keywords:** Computed tomography angiography, Coronary arteries, Dose reduction, Diagnostic performance

## Abstract

To evaluate the effect of radiation dose reduction on image quality and diagnostic accuracy of coronary computed tomography (CT) angiography. Coronary CT angiography studies of 40 patients with (*n* = 20) and without (*n* = 20) significant (≥50 %) stenosis were included (26 male, 14 female, 57 ± 11 years). In addition to the original clinical reconstruction (100 % dose), simulated images were created that correspond to 50, 25 and 12.5 % of the original dose. Image quality and diagnostic performance in identifying significant stenosis were determined. Receiver–operator-characteristics analysis was used to assess diagnostic accuracy at different dose levels. The identification of patients with significant stenosis decreased consistently at doses of 50, 25 and 12.5 of the regular clinical acquisition (100 %). The effect was relatively weak at 50 % dose, and was strong at dose levels of 25 and 12.5 %. At lower doses a steady increase was observed for false negative findings. The number of coronary artery segments that were rated as diagnostic decreased gradually with dose, this was most prominent for smaller segments. The area-under-the-curve (AUC) was 0.90 (*p* = 0.4) at 50 % dose; accuracy decreased significantly with 25 % (AUC 0.70) and 12.5 % dose (AUC 0.60) (*p* < 0.0001), with underestimation of patients having significant stenosis. The clinical acquisition protocol for evaluation of coronary artery stenosis with CT angiography represents a good balance between image quality and patient dose. A potential for a modest (<50 %) reduction of tube current might exist. However, more substantial reduction of tube current will reduce diagnostic performance of coronary CT angiography substantially.

## Introduction

Coronary computed tomography (CT) angiography is increasingly used for non-invasive evaluation of the coronary arteries. With CT, significant coronary artery stenosis can be confirmed or excluded with high accuracy as compared to invasive coronary angiography [1–3]. Current guidelines consider coronary CT angiography appropriate for the evaluation of coronary artery disease in symptomatic patients with low to intermediate pretest probability [4, 5]. However, the relatively high radiation dose associated with coronary CT angiography is of concern [6]. Various technical improvements substantially reduce the radiation dose of coronary CT angiography. Most effective have been the introduction of electrocardiography (ECG)-dependent tube current modulation in helical scans and prospective ECG-triggered acquisitions in axial or helical scans [7, 8]. Furthermore, the use of low kilovoltage (kV) [7, 9] and single-heartbeat full-cardiac imaging by using volumetric acquisition [10, 11] or dual-source imaging protocols [12] may add to reducing radiation dose.

Only two studies reported on the effect of a lower tube current (mA) on the assessment of coronary artery stenosis in coronary CT angiography. One study was performed with a pulsating cardiac phantom [13] and the other study was a patient study [14]. In the phantom study the percentage stenosis of coronary arteries was evaluated; it was concluded that for low dose protocols acceptable image quality was achieved, but with a tendency of overestimating stenosis grade. In the clinical study, a 34 % reduction of the tube current was applied and coronary segments were qualified as either diagnostic or non-diagnostic. For this modest dose reduction, the percentage of segments with diagnostic image quality remained constant at 99 %. A limitation of this latter study is the heterogeneity within the two small patient cohorts. Both studies suggested the potential of dose reduction in coronary CT angiography, but the studies are of limited clinical value since the effect of lowering the tube current on diagnostic accuracy was not studied. Such information is essential in determining whether lower tube current settings may be considered as measure for further dose reduction in coronary CT angiography in clinical practice. Accordingly, the purpose of this clinical study was to evaluate the effect of reduced tube current on image quality and diagnostic accuracy of coronary CT angiography in evaluating coronary artery stenosis.

## Subjects and methods

### Subjects

Institutional review board approval was not required for this retrospective analysis of anonymized data. Coronary CT angiography studies of 40 patients (26 men and 14 women; mean age, 57 ± 11 years) were included. Patients had been scanned on clinical indication with suspicion of coronary artery disease. Twenty patients with significant (≥50 %) coronary artery stenosis and 20 patients without significant coronary artery stenosis were consecutively selected. Inclusion was based on clinical coronary CT angiography reports that explicitly mentioned either “having significant coronary artery stenosis”, or “not having significant coronary artery stenosis”, respectively. Additional selection criteria were sufficient overall diagnostic image quality, 320 mm imaging field of view and optimal image reconstruction at one single cardiac phase.

### Image acquisition

All examinations were performed with a 64-slice multidetector row CT scanner (Aquilion 64, Toshiba Medical Systems, Otawara, Japan). Contrast-enhanced coronary CT angiography acquisitions were obtained; the subsequent reconstructions yielded retrospectively ECG-synchronized scans. Patients with a cardiac frequency prior to the scan exceeding 60 beats per minute received 25–100 mg oral metoprolol when no contra-indications were present. Scan range was planned between the carina and the cardiac apex and scanning was performed in craniocaudal direction. Depending on patient size and expected scan time, 90–120 mL iodinated contrast agent (Iomeron 400 mg/mL, Bracco, Milan) was administered via antecubital vein injection (flow rate 5.0 mL/s), followed by 50 mL saline flush (flow rate 5.0 mL/s). For bolus tracking, a region of interest was placed in the descending aorta and image acquisition was started approximately 7 s after reaching a predefined threshold difference of 100 HU. Scan parameters were 64 × 0.5 mm slice thickness, tube voltage 120 kV (19 patients) and 135 kV (21 patients); tube current between 250 and 440 mA and tube charge between 63 and 112 mAs. Helical pitch ranged from 11.2 to 16.2 (pitch factor 0.18–0.25) and rotation time was 400–500 ms. Tube voltage and tube current depended on patient size and shape as visually estimated by the technician. The helical pitch was optimized automatically for the observed heart rate. Mean heart rate during scanning was 59 (±11) beats per minute.

### Image reconstruction and dose simulation

Images were reconstructed at 0.5 mm section thickness and 0.3 mm increment using a half-scan or multi-segment algorithm. A medium soft-tissue convolution kernel filter was used (FC12). The reconstructed field-of-view (FOV) was 180 mm for all studies. For each patient, the ECG-synchronized datasets were retrospectively reconstructed with the reconstruction window corresponding to the cardiac phase with minimal coronary artery motion; 36 datasets were reconstructed in mid-diastolic phase, 4 datasets during end-systole. In addition to the original clinical reconstruction, four extra reconstructions were made. One additional reconstruction of the original clinical study was made to test intra- and interobserver variability, and three reconstructions were made yielding the low dose simulations representing image quality at 50, 25 and 12.5 % of the dose of the original clinical study.

To create the three reconstructions that simulated the image quality of the coronary CT angiography examinations at lower doses, a validated low dose simulator, developed in MATLAB, was used [15]. This simulator creates the raw scan data (sinograms) that would have been acquired when a lower tube current was applied during the clinical scan. This is achieved by adding noise to the original raw data of the CT scan. The simulated lower dose sinograms were transferred to the CT scanner for image reconstructions. Image noise in the resulting simulated lower dose studies is higher compared to the original study, a phantom study demonstrated that the desired higher noise levels were simulated with an accuracy 3.3 ± 2.6 % for tube currents ranging between 20 and 300 mA [15].

All studies were anonymized and blinded for the associated tube current and dose level. Reconstructed images were transferred to a dedicated workstation for analysis (Vitrea FX, version 1.0, Vital Images, Minnetonka, MN, USA).

### Image analysis

All studies, including the clinical and the simulated CT scans, were analyzed on a workstation with dedicated coronary angiography analysis software (Vital Images, version 1.0, Minnetonka, USA). Image reading was performed independently by two observers. Observer 1 (NB) had 1 year and observer 2 (LK) had 7 years of experience in cardiac CT. Original axial images, coronal and sagittal reconstructions, thin maximum intensity projections and curved multiplanar reconstructions were used for evaluation. Observers were allowed to adapt window width and window level and zoom-factor. Image reading was performed in 10 sessions, each session contained 20 datasets from different patients with different simulated dose levels. Examinations were presented in random order. To prevent recognition bias, at least 1 week interval was applied before the same patient was presented again at a different dose level.

A scoring form was used per dataset to record overall image quality and the presence or absence of coronary artery stenosis. The coronary arteries were evaluated on segmental basis using the 15-segment American Heart Association (AHA) model [16]. As to facilitate locating segments, a map representing the coronary arteries with segment numbers was drawn on the scoring form when the course of the coronary arteries differed from the standard AHA segment classification. First, each segment was graded as being present or absent. If a segment was present, the segment was classified as being diagnostic or non-diagnostic (whether or not the presence and grading of stenosis could be determined reliably). Then, presence of coronary artery disease was evaluated and graded per diagnostic segment by mean luminal diameter reduction in two perpendicular directions as either one of two categories: 1: No significant lumen stenosis (<50 %), or 2: Significant lumen stenosis (≥50 %). For scoring, no distinction was made regarding morhology of stenosis, i.e. calcified or non-calcified lesions. After scoring all segments, overall image quality was evaluated per dataset and classified as 1: Excellent diagnostic image quality, 2: Good diagnostic image quality, 3: Moderate but diagnostic image quality, 4: Limited diagnostic image quality, 5: Non-diagnostic image quality. If applicable, the main factors responsible for restricted diagnostic image quality were noted. Consensus reading was performed after each session for all datasets where interpretation between the observers differed regarding location and/or stenosis grading.

In addition to the observer study, quantitative assessment of image quality was performed. Contrast-to-noise ratio (CNR) measurements were made as previously described [7], and image noise was determined as the standard deviation of Hounsfield unit values in a circular region of interest (1.79 cm^2^) placed in the ascending aorta. CNR was calculated from the difference between the average Hounsfield unit value in the enhanced left ventricular cavity (circular region of interest, 1.79 cm^2^) and the unenhanced left ventricular wall (circular region of interest, 0.81 cm^2^), divided by image noise [7]. It was expected that the effect of dose reduction on image quality would depend on the diameter of the diagnostic coronary artery segments. To be able to assess this effect, the diameters of the segments were measured in the clinical standard of reference images (original 100 % dose study).

### Statistical analysis

Statistical analysis was performed using SPSS version 16.0 for windows (SPSS, Chicago, IL, USA) and SAS statistical package, version 9.2 (SAS Institute Cary, NC, USA). The results from consensus reading were used for further statistical analysis. McNemar tests and paired *t* tests were used when appropriate.

Furthermore, for segment based analysis, a logistic regression model was used to assess differences between dose levels in number of diagnostic segments and segments with ≥50 % stenosis. To adjust for within-patient correlation, a random effect was added to the model. This was to correct for multiple readings for the same patient at distinct dose levels and to correct for multiple stenosis found within a patient. For the patient based analysis, receiver operator characteristics (ROC) analysis was applied to evaluate differences between the simulated dose levels in identifying patients with significant coronary artery stenosis (defined as having at least one coronary artery segment with ≥50 % stenosis). AUCs were compared by evaluating specific points of the ROC-curve for each parameter (i.e. point of specificity at a randomly chosen sensitivity of 80 %). Also, sensitivity, specificity, positive and negative predictive value were calculated. The κ statistic was used to assess intra- and interobserver agreement for significant coronary artery stenosis. Agreement was categorized as poor (κ ≤ 0.20), fair (κ 0.21–0.40), moderate (κ 0.41–0.60), good (κ 0.61–0.80) and very good–excellent (κ 0.81–1.00). A *p* value <0.05 was considered statistically significant.

## Results

### Study details

Mean patient height was 174 ± 11 cm, mean weight 84 ± 18 kg and mean BMI was 27.1 ± 4.1 kg/m^2^ (range: 16.9–36.3). BMI was significantly higher in patients scanned with 135 kV (*n* = 19, mean BMI 30.1 ± 2.9) than in patients scanned with 120 kV (*n* = 21, mean BMI 24.0 ± 2.6, *p* < .001).

The CTDI_w_ was 12.7 ± 2.7 mGy for male patients and 11.9 ± 2.8 mGy for female patients. Mean scan length was 158 ± 20 mm. The dose length product (DLP) was 927 ± 192 mGy cm (men) and 839 ± 217 mGy cm (women). Effective doses correspond to 13.0 ± 2.7 mSv for men and 11.7 ± 3.0 mSv for women when applying conversion factor of 0.014 mSv/mGy cm. Effective doses for 50 % dose correspond to approximately 6.5 mSv for men and 5.9 mSv for women, for 25 % dose to 3.2 mSv for men and 2.9 mSv for women, and for 12.5 % dose to 1.6 mSv for men and 1.5 mSv for women.

### Image quality

**Fig. 1:**
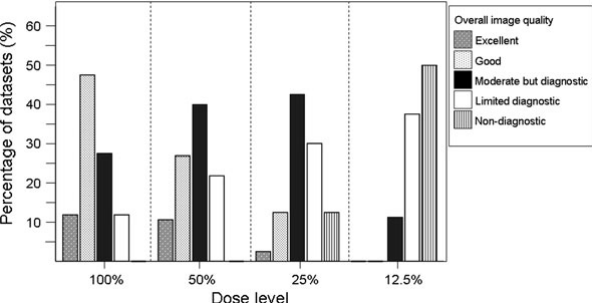
Image quality for all datasets of 40 patients evaluated per dose level. With decreasing dose, overall image quality shifted from predominantly good at 100 % dose to predominantly non-diagnostic at 12.5 % dose. McNemar test revealed significant decrease in image quality for 25 and 12.5 % dose (*p* < 0.001 for both, compared to 100 %)

Figure 1 shows the effect of dose reduction on overall grading of image quality. With decreasing dose, observed image quality shifted from predominantly good and moderate at 100 % dose to limited and non-diagnostic at 25 and 12.5 % dose (*p* < 0.001). However, for 50 % dose, although decrease in image quality was observed, this was not rated as significant (*p* = 0.125). Overall, the main factors responsible for restricted diagnostic image quality reported were: noise (*n* = 108), followed by motion artifacts (*n* = 92), calcifications (*n* = 42) and moderate contrast enhancement (*n* = 11), were with decreasing dose, noise was reported most often as main cause for restricted image quality. Only noise was reported as increasing factor for limited image quality with decreasing dose (in 13 % of readings with 100 % dose, in 58 % with 50 % dose, in 85 % with 25 % dose, and in 100 % with 12.5 % dose). Accordingly, significant decrease in CNR was found with decreasing dose. CNR was 10.5 ± 3.8 with 100 % dose, 7.6 ± 2.9 with 50 % dose, 5.1 ± 1.9 with 25 % dose, and 3.3 ± 1.3 with 12.5 % dose, *p* < 0.0001 for all compared to 100 % dose.

### Image analysis: segment based

**Fig. 2:**
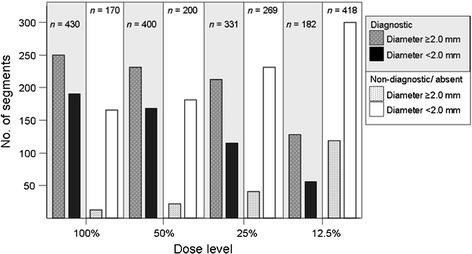
Diagnostic and non-diagnostic image quality for coronary artery segments. Six hundred segments were evaluated per dose level. With decreasing dose, the number of diagnostic segments decreased and the number of non-diagnostic and absent segments increased, compared to the 100 % standard of reference. This was significant for 25 and 12.5 % dose *(p* < 0.0001 for both)

In total, 600 coronary artery segments (i.e. 15 segments in 40 patients) were evaluated per dose level. Figure 2 shows the number of segments graded as diagnostic or as non-diagnostic/absent per dose level and classified by size. Note that diagnostic quality largely depends on coronary artery size ≥2.0 mm; the majority of non-diagnostic segments were smaller than 2.0 mm.

With decreasing dose, the overall number of segments rated as diagnostic (*n* = 430 at 100 % dose), decreased significantly with dose level 25 % (*n* = 331) and dose level 12.5 % (*n* = 182) (*p* < 0.0001 for both). At the dose level of 50 %, 400 segments were rated as diagnostic (93 % of total), but this decrease was not rated as significant (*p* = 0.16). Diameter measurements were obtained in all 430 diagnostic segments; 180 segments (41.9 %) had diameter <2.0 mm and 250 segments (58.1 %) had diameter ≥2.0 mm. For both ≥2.0 mm and <2.0 mm sized segments, a gradual decrease in number of diagnostic segments was observed (Fig. 2). At 50 % dose, the decrease in number of diagnostic segments was not significant for segments ≥2.0 mm (*p* = 0.5), but was significant for segments <2.0 mm (*p* = 0.09). With dose levels of 25 and 12.5 %, this was significant for both (both *p* ≤ 0.0002).

Table 1 shows per coronary artery segment the coronary artery diameter and diagnostic score per dose level. As can be observed, decrease in diagnostic score was found at lower dose and this effect was more prominent for smaller (more distally located) coronary artery segments.

**Table 1:** Number of patients where distinctive coronary artery segments were scored as diagnostic per dose level

	Mean diameter (mm)	100 %	50 %	25 %	12.5 %
1. Proximal RCA	3.0 ± 0.8	35	35	33	18
2. Mid RCA	2.8 ± 0.8	35	34	30	19
3. Distal RCA	2.6 ± 0.7	33	33	27	16
4. Right PDA	1.3 ± 0.5	23	19	15	8
5. Left main	3.7 ± 0.8	39	37	34	24
6. Proximal LAD	3.0 ± 0.6	38	38	33	21
7. Mid LAD	2.5 ± 0.5	36	35	27	15
8. Distal LAD	1.7 ± 0.4	34	32	24	14
9. 1st diagonal	1.4 ± 0.5	33	30	23	14
10. 2nd diagonal	1.1 ± 0.4	30	21	18	7
11. Proximal Cx	2.2 ± 1.0	35	36	29	16
12. Mid Cx	1.4 ± 0.6	28	22	21	6
13. Obtuse marginal	1.5 ± 0.8	21	22	14	3
14. Posterolateral	1.5 ± 0.8	8	5	2	1
15. Left PDA	0.8 ± 0.5	2	1	1	0

Datasets of 40 patients were evaluated per dose level. Missing segments are partly due to non-diagnostic quality and partly due to the absence of segments

### Image analysis: patient based

**Fig. 3:**
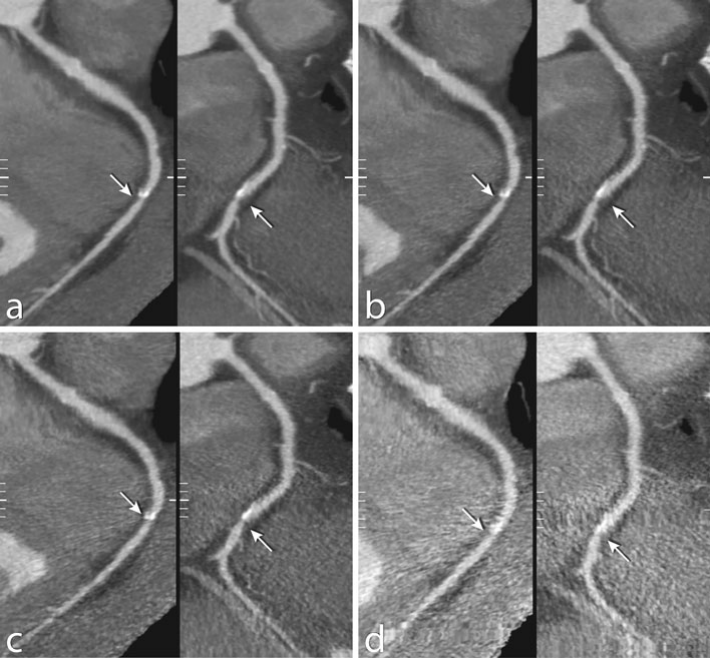
Low dose simulations of coronary CT angiography in a 64-year old male. Images show curved multiplanar reconstructions of the right coronary artery for **a** 100 % dose, **b** simulated 50 % dose, **c** 25 and **d** 12.5 % dose. A significant stenosis in the mid part of the right coronary artery (segment 3) was found (*arrows*) with 100 and 50 % dose. With 25 and 12.5 % dose, the stenosis was classified as “not significant”

Table 2 shows classification of patients with significant stenosis for each simulated dose level. The number of patients classified with significant coronary artery stenosis decreased consistently, the decrease was significant at 25 and 12.5 % dose (*p* ≤ 0.02), and not significant at 50 % dose (*p* = 0.55). With 12.5 % dose, only 5 out of 20 patients were recognized as having significant coronary artery stenosis. The effect of dose reduction resulted mainly in underestimation of the number of patients with significant coronary artery stenosis (increase of the number of false negative scores) but had less effect on the number of false positive scores. This is illustrated by a case example shown in Fig. 3. Note that diagnostic image quality is preserved at 50 % dose, whereas further increase in noise for dose reduction down to 25 and 12.5 % dose hampers diagnosis. No significant differences were found in the identification of coronary artery stenosis between patients scanned with 120 kV and those scanned with 135 kV (*p* = *0*.7).

**Table 2:** Patient evaluation: Number of patients identified with at least one coronary artery segment with ≥50 % stenosis versus those without significant stenosis per dose level, compared to 100 % dose

	100 % dose ≥50 % stenosis (*n* = 20)	No stenosis (*n* = 20)	*p*
50 % dose			
Stenosis	18	2	
No stenosis	2	18	0.55
25 % dose			
Stenosis	11	3	
No stenosis	9	17	.002*
12.5 % dose			
Stenosis	5	1	
No stenosis	15	19	<.0001*

* represents *p*-value < 0.05

Table 3 shows the predictive values of the 50, 25, and 12.5 % doses in identifying patients with significant coronary artery stenosis compared to the 100 % dose. Compared to the 100 % dose, accuracy and AUC for depicting significant coronary artery stenosis decreased with decreasing dose, AUC at 50 % dose was 0.90 (not statistically significant difference, (*p* = 0.4)) and the decrease was statistically significant for 25 % (AUC 0.70) and 12.5 % dose (AUC 0.60), both *p* < 0.0001;

**Table 3:** Overall diagnostic performance of simulated low dose coronary CT angiography in identifying significant (≥50 %) coronary artery stenosis on patient basis compared to standard of reference 100 % dose

Dose (%)	Sensitivity	Specificity	PPV	NPV	Accuracy	AUC
50	90 (67–98)	90 (67–98)	90 (67–98)	90 (67–98)	90 (81–99)	0.90 (0.79–1.0)
25	55 (32–76)	85 (61–96)	79 (49–94)	65 (44–82)	70 (56–84)	0.70 (0.53–0.87)
12.5	25 (1–49)	95 (73–100)	83 (36–99)	56 (38–72)	60 (45–75)	0.60 (0.42–0.78)

Data in parenthesis represent upper and lower bound 95 % confidence interval. *PPV* positive predictive value, *NPV* negative predictive value, *AUC* area-under-the-curve

### Intra- and interobserver agreement

Table 4 shows the intra- and interobserver agreement per dose level. Good to excellent intraobserver agreement was found for detecting ≥50 % stenosis on segmental basis with κ-values of 0.68 (observer 1) and 0.75 (observer 2). Patient-based intraobserver agreement was moderate for observer 1 (κ-value 0.60) and very good–excellent for observer 2 (κ-value 0.85). Interobserver agreement was good for segment-based analysis and moderate for patient-based analysis with κ-values of 0.73 and 0.59, respectively. Overall, with decreasing dose levels intraobserver and interobserver agreement decreased.

**Table 4:** Intra- and interobserver agreement for identifying significant coronary artery stenosis per dose level

	Observer 1 (κ)	Observer 2 (κ)	Interobserver (κ)
*Segment based*			
100 % versus 100 %	0.68	0.75	0.73
100 % versus 50 %	0.65	0.71	0.71
100 % versus. 25 %	0.65	0.69	0.65
100 % versus 12.5 %	0.52	0.60	0.68
*Patient based*			
100 % versus 100 %	0.60	0.85	0.59
100 % versus 50 %	0.54	0.69	0.54
100 % versus 25 %	0.54	0.42	0.35
100 % versus 12.5 %	0.05	0.21	0.05

## Discussion

The main finding of the present study was that the identification of patients having significant coronary artery stenosis decreased consistently at doses of 50, 25 and 12.5 % of the standard clinical acquisition (100 %). The effect was relatively weak at 50 % of the dose, and was strong at dose levels of 25 and 12.5 %. At lower doses an increase for false negative findings was observed. Furthermore, the number of coronary artery segments assigned as being of diagnostic quality decreased, as well as the number of identified significant coronary artery stenosis. We have also shown that reliability and reproducibility for detecting significant coronary artery stenosis deteriorated at lower dose, as demonstrated by decreased inter- and intraobserver variability. The effect of reduced image quality at lower doses was confirmed by the results of measurement of the CNR; the observed effect of dose on CNR was in accordance with the theoretically expected relationship.

Information regarding the effect of reducing the tube current on image quality is sparse in coronary CT angiography, but is essential for adequately balancing patient dose and image quality [13, 14]. One study investigated the assessment of the degree of stenoses by using a dynamic cardiac phantom in relation to different tube currents (650, 550, 450 and 350 mA; with 120 kV) [13]. In that study, image quality was found acceptable for all tube current settings. In addition, no significant differences were found in diagnostic accuracy, determined by comparing measured stenosis areas with physical sizes and number of stenosis in the simulated vessels, although low-dose protocols showed tendency towards overestimating stenosis [13]. However, only simulated, large diameter (>3 mm) vessels were used and no calcified plaques were present. Moreover, the heart rate of the cardiac phantom was relatively low (55 bpm). The design of the phantom study does not reproduce relevant clinical conditions, and is therefore of limited value. Another study investigated the effect of lowering tube current time product from 330 to 220 mAs in a prospective study for 40 patients with a BMI below 25. That study reported 34 % dose reduction while no significant difference was found in observed number of diagnostic segments [14]. Although the number of diagnostic segments was 99 % in that study, the accuracy in detecting stenosis was not evaluated and no reference standard was available. Also, all patients had BMI below 25, and the effects on diagnostic image quality may be more obvious in patients with higher BMI. Therefore, it is unclear what the effect of lowering tube current time product was on diagnostic accuracy for that study.

In our study, clinical scans of patients were used to investigate the effect of lowering tube current settings on image quality and diagnostic accuracy. Different simulated low dose acquisitions were created from the same clinical examination. By using a low dose simulator, all patient factors such as heart rate, coronary artery anatomy, and BMI as well as other examination factors were kept constant. As a result, the change in diagnostic accuracy, image quality and CNR found in our study must be considered attributable to the effect of decreased tube current alone. Also, our 100 % reference value radiation dose was comparable to that reported in other studies that used 64-slice coronary CT angiography with retrospective ECG-gating [2, 8, 9, 17]. In those studies, coronary CT angiography doses ranged between 12 and 21 mSv. In our study, the 100 % clinical reference dose of 12–13 mSv was even at the lower end of this range, indicating that the acquisition protocol that was used in our study was appropriately optimized for patient dose. Also, no large differences were found in other factors influencing patient exposure (i.e. scan length, BMI and heart rate during scanning) [17, 18]. We therefore consider the patient examinations used in our study representative for good clinical practice with 64-slice retrospective ECG-gating coronary CT angiography techniques.

The technique of retrospective ECG gated reconstructions that was used in this current study is associated with a relatively high effective dose since the very wide acquisition window includes several full R–R intervals and the acquisition is performed with a constant tube current. For retrospective gated reconstructions the selection of the reconstruction window (i.e. the cardiac phase with least motion artefacts) occurs during the reconstruction. Several CT technologies that allow for dose reduction are available. Such technologies include ECG triggered tube current modulation, and prospective ECG-triggered acquisitions. Other measures such as optimizing scan parameters (i.e. scan range, acquisition window, tube voltage and patient preparation) and reconstruction algorithms (iterative reconstruction) have also shown to substantially reduce radiation dose, without significant decrease in image quality [18].

ECG-triggered tube current modulation is a technique that prospectively reduces the tube current outside the reconstruction window, during the reconstruction window the tube current remains unchanged. With ECG-triggered tube current modulation patient dose can be reduced, but the reconstruction window has to be established in advance and cannot be modified during the reconstruction. With prospective ECG-triggered acquisitions the acquisition window becomes much smaller because the tube current is only switched on during the cardiac rest phase and tube current is completely switched off during the remainder of the cardiac phase. With prospective ECG-triggered acquisitions, compared to tube current modulation, patient dose can be reduced even further. The dose reduction that can be realized with ECG-triggered tube current modulation (up to 30 %) or prospective ECG-triggered acquisitions (up to 70 %) does not have an effect on the cardiac phase that is used for reconstruction of the coronary CT angiograms [8, 19]. Although implementation of such new dose saving technologies is preferred above reduction of tube current, this may also be used as additional measure to reduce radiation dose in prospective ECG-triggered acquisitions.

Good but not perfect intra-individual variation may be explained by well-recognized difficulties in grading coronary artery stenosis by CT (especially for intermediate-grade stenoses or when calcified plaque is present), that was performed in combination with a dichotomous decision for assigning either a stenotic or non-stenotic value. The moderate to good interobserver agreement was likely influenced by differences in cardiac CT experience level between both observers. Diagnostic performance has been shown to improve with increasing experience [20], as was also the case in our study.

Our study had some limitations. In this study, a relative small group of 40 patients was used for analysis and patients were selected 50/50 based on the presence or absence of coronary artery stenosis. It is not known what the effect of reducing tube current settings would be in a large, unselected population. In our analysis, plaque composition and size were not taken into account. Also, no distinction was made between plaque composition, whereas calcifications were among factors for restricted image quality. It is not known what the effect of increased image noise would be on this parameters.

In conclusion, the presented clinical acquisition protocol for evaluation of coronary artery stenosis with CT angiography represents a good balance between image quality and patient dose. A slightly lower tube current (up to 50 % lower) may be used in clinical coronary CT angiography acquisitions as measure to limit radiation dose, but a more substantial reduction of tube current will reduce diagnostic performance of coronary CT angiography to an unacceptable level.
